# Dengue-Related Ocular Complications: Spectrum, Diagnosis, and Management

**DOI:** 10.3390/pathogens14090872

**Published:** 2025-09-02

**Authors:** Jiaxin Deng, Yaru Zou, Mingming Yang, Jing Zhang, Zizhen Ye, Yuan Zong, Kyoko Ohno-Matsui, Koju Kamoi

**Affiliations:** 1 Department of Ophthalmology & Visual Science, Graduate School of Medical and Dental Sciences, Institute of Science Tokyo, Tokyo 113-8510, Japan; dengjiaxin.med@gmail.com (J.D.); alicezouyaru519@gmail.com (Y.Z.); yangmm-12@outlook.com (M.Y.); zhangj.c@foxmail.com (J.Z.); yezizhen518@gmail.com (Z.Y.); zongyuan666@gmail.com (Y.Z.); k.ohno.oph@tmd.ac.jp (K.O.-M.); 2 Department of Ophthalmology, Zhongshan Torch Development Zone People’s Hospital, Zhongshan 528436, China

**Keywords:** dengue fever, maculopathy, uveitis, hemorrhage, retinal vasculitis

## Abstract

Dengue virus infection frequently involves the eye, manifesting with hemorrhages, uveal inflammation, retinal vascular changes and maculopathy. These ocular manifestations may arise during the acute febrile phase or emerge weeks later. Studies from endemic regions report that up to one-quarter of hospitalized patients develop eye-related symptoms. Furthermore, studies confirm a higher risk of new uveitis cases following dengue infection. Breakdown of the blood–ocular barrier—driven by antibody-mediated enhancement, complement activation and release of inflammatory mediators—leads to vascular leakage, tissue injury and ischemia. Diagnosis relies on clinical examination supplemented by imaging (OCT, angiography) and laboratory confirmation of dengue. Mild anterior inflammation often responds to topical steroids, while sight-threatening posterior disease requires systemic corticosteroids and, in refractory cases, immunomodulatory agents. Visual outcomes depend on the initial severity; anterior uveitis typically resolves without sequelae, whereas vasculitis or foveal involvement may leave lasting deficits. This review integrates the current understanding of dengue-related eye disease, emphasizing its varied presentations and the importance of early recognition. Further research into targeted, mechanism-based therapies is needed to optimize visual outcomes.

## 1. Introduction

Dengue, a systemic viral infection, is of increasing international concern, with growing incidence putting immense pressure on healthcare systems because of climate change, population growth, human mobility, and urbanization. Dengue is currently endemic in more than 100 countries in tropical and subtropical regions of southeast Asia, Africa, the west Pacific, and the Americas [1]. Dengue is also the most commonly reported arboviral disease among travelers, showing a rising global trend between 1995 and 2020 [2]. There are an estimated 58.40 million symptomatic dengue virus infections (95% uncertainty interval [95% UI] 24 million–122 million) annually, costing US$8.9 billion (95% UI 3.7 billion–19.7 billion), bringing important economic impact [3].

Dengue viruses are primarily transmitted through the bite of an infected mosquito vector, with *Aedes aegypti* and *Aedes albopictus* as the predominant vectors [4]. Dengue viruses are single-stranded RNA viruses of the genus Flavivirus in the family Flaviviridae. They are classified into four serotypes—DENV-1, DENV-2, DENV-3, and DENV-4—due to their differing interactions with human antibodies. The four dengue virus serotypes share approximately 65% of their genomes, but some genetic variations exist even within the same serotype [4,5]. Infection with a single dengue virus serotype provides lifelong immunity to that specific serotype. When an individual previously infected with one serotype becomes infected with a different serotype, the condition is referred to as secondary dengue [6].

The clinical spectrum of dengue ranges from a febrile illness to life-threatening dengue shock syndrome, and it is often accompanied by classical symptoms of severe headache, retro-orbital pain, myalgia and arthralgia, macular or maculopapular rash [7,8]. Clinicians should consider dengue in febrile patients with a history of residence in, or travel to, areas with documented ongoing transmission within the two weeks preceding symptom onset [7,9].

Ocular involvement is a relatively common manifestation of dengue virus infection. Most ocular manifestations appear around seven days after the onset of fever [10,11,12], although uveitis can occur 3–5 months after dengue infection, with no other identifiable cause for the condition [13]. A systematic review reported an overall prevalence of ocular complications in dengue fever at 23.99%, with uveitis occurring in 18.72% of all patients [14]. Moreover, according to a cohort study involving 173,324 person-years of dengue cases and 709,498 person-years in a control cohort, dengue infection may increase the risk of uveitis [15].

This review focuses specifically on the ocular manifestations of dengue infection. We aimed to systematically summarize the clinical manifestations, diagnostic tests, treatment methods, and potential mechanisms of dengue-related eye diseases—with the goal of providing a theoretical basis for early diagnosis and appropriate clinical management.

## 2. Classification and Clinical Course of Dengue Systemic Illness

Dengue is an acute febrile illness that is self-limiting and presents with non-specific symptoms. The 1997 World Health Organization (WHO) guidelines classified symptomatic dengue virus infection into three categories: dengue fever, dengue hemorrhagic fever (DHF), and dengue shock syndrome (DSS). DHF is defined by the presence of fever, bleeding tendency, thrombocytopenia (≤100,000 platelets/mm^3^), and signs of plasma leakage. It is further divided into four grades, with Grades III and IV—marked by circulatory failure—classified as DSS. Most of the published studies on dengue-related eye disease have relied on this dengue classification system. However, in the revised 2009 WHO guidelines, symptomatic dengue is categorized as dengue without warning signs, dengue with warning signs, or severe dengue [8].

Symptomatic dengue typically progresses through three clinical phases: febrile, critical, and recovery. The febrile phase usually lasts 2 to 7 days and is marked by the sudden onset of high-grade fever (≥38.5 °C), often accompanied by nausea, vomiting, a transient macular rash, aches, and other constitutional symptoms. In most cases, the fever subsides and is followed by the recovery phase; such instance would be categorized as uncomplicated dengue [8].

A subset of dengue patients would enter the critical phase, typically around days 4 to 6 of illness, often coinciding with the decline of fever [8]. The defining feature of severe dengue during this phase is plasma leakage, where protein-rich fluid escapes from the blood vessels into surrounding tissues, potentially resulting in shock and occasionally accompanied by bleeding [16,17]. Patients who begin to improve and recover are classified as having dengue with warning signs. However, in about 2–5% of cases, the illness may progress to severe dengue [18].

Severe dengue is diagnosed based on specific criteria, which include: significant plasma leakage resulting in shock or fluid buildup causing respiratory distress; major bleeding as assessed by a clinician; and serious organ involvement—such as of the central nervous system, heart, or liver (indicated by an aspartate aminotransferase or alanine aminotransferase concentration of 1000 IU/L or more) [8].

## 3. Risk Factors for Dengue-Related Ocular Disease

Gender and ethnicity are not recognized as established risk factors for Dengue-related ocular complications [19]. Although several reports suggest that ocular manifestations of dengue are more prominent in males, other studies indicate a higher diagnostic rate in females [20,21,22,23]. In larger case series, the average age at presentation typically falls between 28.7 and 41.4 years [24,25]. A cohort study further reported that patients aged 60 years and older have a significantly higher risk of developing dengue-associated uveitis compared to those aged 40 to 59 years [15]. Additionally, individuals with a history of stroke or transient ischemic attack who were diagnosed with dengue were more likely to develop uveitis than those without dengue infection [15].

Specific dengue virus serotypes may increase the risk of developing dengue-related eye disease. Chee et al. reported that maculopathy was observed in 10% of patients in the 2005 group, when DENV-1 was predominant, but in none of the 87 patients from the 2007 group, which was dominated by serotype 2 (*p* = 0.002) [25]. Notably, on Réunion Island, the predominant dengue serotype shifted from DENV-2 to DENV-1 in 2020, with DENV-1 becoming the only detected serotype in 2021. During the same period, 126 cases of dengue-associated maculopathy were reported for the first time [22]. Compared to DENV-2, outbreaks of dengue fever (DF) predominantly caused by DENV-1 are associated with a higher prevalence of color vision impairment, cotton wool spots, hemorrhagic tendency, and liver function abnormalities [26]. Besides serotype differences, specific viral strains may also be linked to disease manifestations [27,28].

Leukopaenia and hypoalbuminaemia are significant risk factors for the onset of ocular symptoms [29]. These factors may, respectively, increase susceptibility to opportunistic ocular infections and lead to heightened vascular permeability [29,30,31]. Ocular complications may arise as a result of DENV infection, with thrombocytopaenia potentially contributing indirectly to retinal hemorrhages [23,32].

## 4. Pathogenesis

The mechanisms underlying ocular complications in dengue fever are unknown but are commonly thought to be immune-mediated. The major reason is that most ocular manifestations appear around seven days after the onset of fever [10,11,12] while reports of uveitis occurring 3–5 months after dengue infection, in the absence of other identifiable causes, provide further support for this [13].

Immune complex deposition, complement activation, and autoantibody formation have been postulated to be implicated. Antigen–antibody complexes may activate the classical complement pathway, leading to C3 and C4 consumption and formation of membrane attack complexes [19,33]. A transient reduction in C4 levels observed in several patients, as well as the occlusive vasculopathy seen in one-third of eyes in this series, could also be explained by immune complex deposition [33], although one study did not demonstrate a significant correlation [19]. The dengue virus itself may act as an antigenic trigger for autoimmune responses against ocular tissues, including the retina, retinal pigment epithelium, choroid, and corneal endothelium [34]. And the formation of autoantibody against corneal endothelial cells potentially contribute to stromal keratitis [35]. In parallel, transient vascular leakage—driven by viral invasion of endothelial and antigen-presenting cells, T-cell activation, and the release of vasoactive cytokines such as interleukins (ILs) and tumor necrosis factor-alpha (TNF-α)—further amplifies endothelial injury [36]. Disruption of the blood–ocular barrier thereby facilitates the entry of inflammatory mediators into ocular tissues, resulting in anterior uveitis and periphlebitis [34].

## 5. Clinical Features of Dengue Eye Disease

### 5.1. Subconjunctival Hemorrhage

Subconjunctival hemorrhage (SCH) is a common ocular feature of dengue infection, which may present unilaterally or bilaterally [37]. Subconjunctival hemorrhage is typically self-limiting and resolves spontaneously, as the extravasated blood is gradually reabsorbed within a few days to two weeks. Clinically, SCH presents as a painless, diffuse, and petechial hemorrhage beneath the conjunctiva [37].

The role of thrombocytopaenia in SCH is still unclear. A prospective cohort study reported a 37.3% incidence of subconjunctival hemorrhage, with 90% of affected patients having platelet counts below 50,000/μL [37]. However, in a prospective study, SCH was reported as the most common early sign in dengue patients who had not yet been diagnosed and had platelet counts above 50,000/μL [38]. And in a study involving 50 patients with platelet counts ranging from 13,000 to 40,000/μL, 8% developed SCH [34]. Furthermore, dengue non-structural protein 1 (NS1) viral proteins also play a role in vascular events [39]. NS1 has been implicated in vascular leakage, and its levels closely correlate with platelet counts in DHF/DSS, as shown in both in vitro and in vivo models [40].

### 5.2. Uveal Inflammation

#### 5.2.1. Dengue Uveitis

The exact pathogenesis of dengue uveitis is unknown but is commonly believed to be immune-mediated, especially as uveitis often arises 3–4 months after resolution of systemic symptoms [13]. A cohort study showed that a significantly higher cumulative incidence of uveitis in patients with dengue fever compared to non-dengue cohort [15]. Furthermore, Wu et al. found an increased risk of uveitis with advancing age [15]. They hypothesized that aging may lead to a gradual decline in immune self-regulation, which could increase the susceptibility to uveitis following Dengue fever infection, but further fundamental research is needed to confirm this hypothesis [15]. A meta-analysis estimated that uveitis occurs in approximately 18.7% of dengue patients, underscoring its importance as a complication [14].

Among dengue-related uveitis cases, anterior uveitis is relatively uncommon with an estimated incidence of 4.8–17% among patients with ocular involvement [41]. Blurring vision is the most common symptom, and the condition is usually painless. It is noteworthy that a clinical study showed that even in cases of severe anterior segment inflammation, ciliary congestion was minimal or absent, which may lead clinicians to underestimate the severity of the inflammation or encounter difficulties in diagnosis [13]. The keratic precipitates ranged in size from fine to medium to large and were diffusely distributed across the cornea in nearly all cases [13].

Intermediate uveitis, predominantly found in the vitreous and along the peripheral retina, is quite rare. It was observed in 3.2% to 12.3% of dengue patients presenting with ocular symptoms [24,42]. And among patients with dengue-associated maculopathy, 31% of patients had vitritis and 11% had anterior and intermediate uveitis [33].

Posterior uveitis, including retinitis, chorioretinitis and neuroretinitis, is relatively common among ocular complications of dengue infection. A study including 65 eyes with dengue infection found that 15 eyes (23.1%) exhibited retinal vasculitis, 13 eyes (20%) showed extensive panretinal vasculitis with exudative retinal detachment, and 7 eyes (10.8%) demonstrated posterior vitreous cells [24]. In a retrospective study, posterior uveitis or retinal vasculitis was reported as the most common presentation, with blurred vison being the leading symptom (n = 41, 75.9%), followed by floaters (n = 9, 17.0%), scotoma (n = 5, 9.3%), and metamorphopsia (n = 3, 5.7%) [20]. An experimental study showed human retinal pigment epithelial cells (RPE) and human retinal endothelial cells can be infected by DENV [27], and human retinal pigment epithelial cell monolayers showed significant upregulation of genes involved in the type I interferon response [43]. This finding suggests that direct viral infection may play a role in the pathogenesis of posterior uveitis. Inflammation and ischemia are postulated to be mechanisms of retinal tissue damage, as evidenced by features such as vitreous inflammatory cells, cystoid retinal edema, and deep capillary plexus ischemia. The overlapping contribution of these mechanisms is reflected in structural changes like retinal hemorrhage, outer retinal thinning, and persistent scotomas [23].

#### 5.2.2. Choroiditis

Choroiditis, characterized by yellow-white lesions distributed around the papillomacular bundle and fovea, may present as focal or multifocal chorioretinitis frequently associated with macular involvement [19]. Dry atrophic perifoveal pigmentary changes may develop following an episode of inflammatory chorioretinitis, often appearing as nummular-shaped pigmented scars [44]. A case of secondary dengue infection documented a retinitis lesion approximately 2-disc diameter in size, accompanied by retinal vasculitis and serous macular detachment. Optical coherence tomography (OCT) revealed inner retinal thickening, increased hyperreflectivity, disorganization of the retinal layers, and fine dot-like deposits beneath the internal limiting membrane (ILM) [45].

#### 5.2.3. Optic Neuritis

Optic neuritis is generally bilateral and often coexists with dengue maculopathy [46,47]. Clinical features of optic neuritis include decreased visual acuity and often coexists with dengue maculopathy [46]. Fundus examination may show optic disc edema characterized by blurred disc margins, disc hyperemia, and associated vascular changes such as vessel engorgement and hemorrhages [47,48].

#### 5.2.4. Henle Fiber Layer Hemorrhage

Henle fiber layer hemorrhage was reported in a 19-year-old man, with paracentral acute middle maculopathy. Dilated fundoscopic examination revealed a small deep hemorrhage near the fovea, and OCT revealed a petaloid shape hyperreflectivity involving both outer nuclear and plexiform layers [49].

### 5.3. Retinal Vasculitis

**Figure 1:**
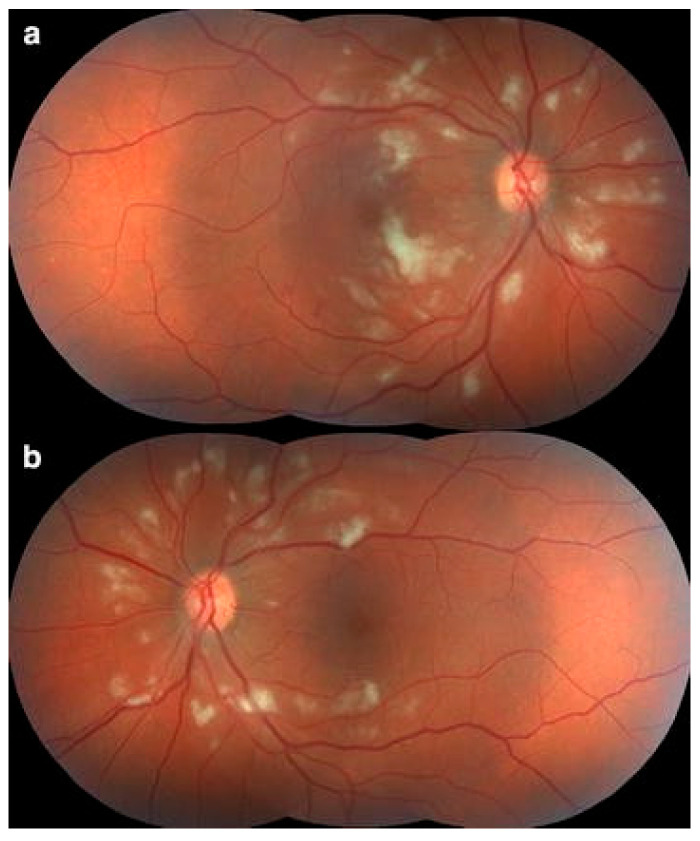
Fundus photographs of the right (**a**) and left (**b**) eyes in a patient with presumed dengue-related retinitis show cotton-wool spots and retinal hemorrhages. (Adapted with permission from [50] 2017 de Andrade et al.).

Among patients with dengue-related ocular complications, 30.7% were found to have retinal vasculitis [10], and 18.1% of those with dengue-associated maculopathy, most of which were extensive and accompanied by exudative retinal detachment [33]. It often coexists with retinitis or retinochoroiditis and may lead to complications such as macular edema or exudative retinal detachment [44,45]. The most frequently observed clinical sign was perivascular sheathing, affecting both venules and arterioles—though venular involvement was more prevalent [12,33]. Additional findings included retinal hemorrhages, cotton wool spots, and perivascular exudates (Figure 1) [11,12,44]. On fluorescein angiography (FFA), characteristic features included early vascular blockage or delayed perfusion, followed by mid- and late-phase vessel wall hyperfluorescence, knobby hyperfluorescence of capillaries, and localized areas of blocked fluorescence [11,12,33]. DENV infection has also been shown to upregulate leukocyte adhesion molecules in retinal endothelial cells and to disrupt intercellular junctions in pigment epithelial cells, providing possible mechanisms for vascular leakage [27].

### 5.4. Anterior Segment Implications

Anterior segment implications are rare but important. In a case series of 29 eyes, 10% presented with isolated corneal or scleral melting attributed to DHF and 35% of patients have thrombocytopaenia [51]. A study presented a 25-year-old woman with stromal keratitis, occurring 12 days after being hospitalized due to fever. However, this onset of ophthalmic complications does not correlate with low platelet count [35]. Furthermore, Kamoi et al. reported a case of dengue-associated scleritis with no recurrence of active inflammation over 18-years, though progressive scleral thinning continued [52]. Although the exact mechanisms underlying dengue-related corneal and scleral damage remain unclear, the clinical cases suggest the possibility of long-term, immune-mediated processes.

Acute angle-closure glaucoma (AACG) has also been reported in dengue infected patients, which suggested that inflammation may induce structural changes within the eye. Angle closure occurs through forward displacement of the lens–iris complex, leading to a decreased anterior chamber depth [53].

### 5.5. Maculopathy

Dengue-related maculopathy (DRM) is one of the most common ocular manifestations of dengue fever and encompasses a spectrum of structural alterations in the macula. Among these, macular edema is the most frequently reported ocular sign [33], while other findings include intraretinal hemorrhages, white or yellow retinal spots, and exudates [19]. A cross-sectional observational study reported a 10% prevalence of DRM among dengue patients [19]. Based on OCT findings, Teoh et al. classified DRM into three subtypes: Type 1 (diffuse retinal thickening), Type 2 (cystoid macular edema), and Type 3 (“foveolitis” with disruption of outer photoreceptor layers or RPE) [24]. Although visual acuity generally improves over time, persistent scotomas were noted in all Type 3 cases and more than half of Type 2 cases [24].

Visual symptoms associated with maculopathy typically emerge approximately 7 days after the onset of fever [33]. In one study, about 24.1% of hospitalized dengue patients experienced visual disturbances [19]. The most commonly reported symptoms were blurred vision (51.2–87%) and scotomas (29.1–63%), with floaters being less frequent (1%) [19]. Other visual abnormalities, such as micropsia and metamorphopsia, were also occasionally reported [13,18,34].

A two-year follow-up study demonstrated gradual visual improvement in most patients, with only 2.7% having a final visual acuity worse than 20/80. However, visual outcomes varied among DRM subtypes; persistent scotomas were reported in 100% of Type 3 cases and 56% of Type 2 cases during the follow-up period [24].

Agarwal et al. introduced the term “Dengue-induced ischaemic foveolitis and outer maculopathy” (DIIFOM) to describe a spectrum of maculopathy involving predominantly ischaemic and inflammatory changes in the outer retina. SS-OCT findings for DIIFOM include vitreous cells, hyperreflectivity of the outer plexiform layer, and a unique “conical” elevation of retinal layers. OCTA reveals deep capillary ischemia in all cases, with about half also showing superficial capillary disruption. Systemic corticosteroid treatment improves vitreous inflammation and retinal integrity, yet persistent retinal plexus flow deficits, significantly correlated with baseline visual acuity, suggest that DIIFOM’s mechanism involves both ischemic and inflammatory processes [23]. DRM could also severely affect patients with long-term scotoma [23,24,54]. In a case series, 55.6% of patients with dengue maculopathy manifested acute macular neuroretinopathy (AMN) and persisted with visual scotomas after 6 months of follow-up [55].

**Figure 2:**
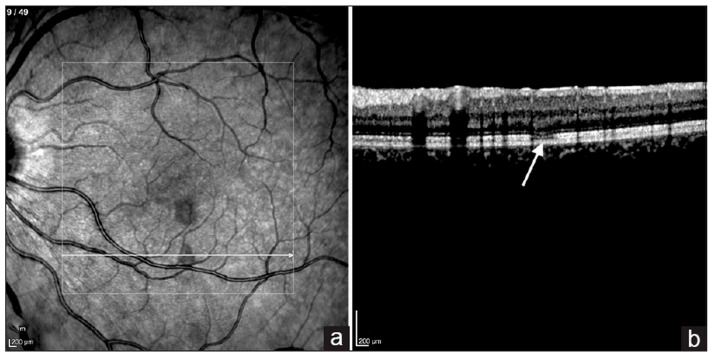
A 27-year-old woman developed AMN following dengue infection. (**a**) Infrared reflectance imaging of the left eye shows two oval-shaped hyporeflective lesions. (**b**) Spectral-domain optical coherence tomography (SD-OCT) horizontal scan across the oval-shaped hyporeflective lesion demonstrating attenuation of the ellipsoid zone. The arrow indicates the hyporeflective lesion, and the frame highlights the area of ellipsoid zone attenuation.(Adapted with permission from [57] 2024 Wang et al.).

AMN is a rare manifestation of maculopathy, by the onset of sudden paracentral scotoma with wedge-shaped dark reddish-brown macular lesions [56]. Infrared reflectance imaging revealed focal hyporeflective regions within the macular area (Figure 2) [57]. Disruptions of the ellipsoid zone, external limiting membrane, and interdigitation zones could be detected by OCT [56,58]. Further clarity comes from en-face OCT angiography (OCTA), which demonstrates significant flow deficits and disruption within both the superficial and deep capillary networks of the retina, particularly in the foveal region. This often accompanies an enlargement of the foveal avascular zone and a distinctive “hairpin loop” pattern in neighboring retinal capillaries, all pointing to retinal capillary ischemia [58]. The proposed mechanism underlying AMN involves choriocapillaris ischemia, potentially caused by immune complex deposition resulting in endothelial injury or obstruction of collecting venules [56].

### 5.6. Panophthalmitis

Panophthalmitis is rarely reported but is one of the most severe types of ocular infectious diseases. A study indicated that it could occur in a notable 26.07% of dengue-infected patients who develop ocular manifestations [21]. Maan et al. highlighted that early systemic antibiotic treatment and surgical intervention are essential in managing dengue-associated panophthalmitis, although visual outcomes remain poor despite such efforts [21].

A plausible pathogenesis of dengue-associated panophthalmitis involves secondary endogenous endophthalmitis resulting from dengue-induced sepsis. Microorganisms may cross the blood–ocular barrier, localize within the uveal or retinal circulation, and form septic foci in retinal capillaries, potentially spreading into the vitreous cavity [59]. The detection of dengue virus RNA in limbal tissues from donors with a history of viral hemorrhagic fever further supports the possibility of direct viral invasion of ocular structures [60].

## 6. Investigations

### 6.1. Laboratory Diagnosis of Dengue

Accurate and timely laboratory diagnosis of dengue is crucial for effective management. For the first 7 days of illness, a combination of tests on whole blood, plasma, or serum is recommended: either a nucleic acid amplification test (NAAT) and an IgM antibody test, or an NS1 enzyme-linked immunosorbent assay (ELISA) test and an IgM antibody test [7]. In regions with co-circulating arboviruses (e.g., Zika, chikungunya), serologic cross-reactivity can reduce specificity; when feasible, confirm equivocal or unexpected serologic results with virus neutralization testing or NAAT to improve diagnostic certainty [7]. Dengue virus RNA can usually be detected by NAAT during the first 7 days of illness, with serum specimens being the most extensively validated and preferred sample for testing [61]. After the first week, when the viral load typically decreases, the IgM ELISA antibody test becomes the primary recommendation, as IgM antibodies remain reliably detectable for approximately three months [62]. Distinguishing between primary and secondary dengue infections is also important for prognosis and management [6,63]: IgG/IgM ratio greater than 1.10 at an early stage of the clinical course demonstrates excellent diagnostic performance, showing 100% sensitivity, 97.4% specificity, and 67.5% accuracy in identifying secondary infections [63].

### 6.2. Amsler Grid Testing, Microperimetry and Visual Field Assessment

For patients with dengue-related maculopathy, a comprehensive assessment of visual function is essential. Amsler grid testing is a valuable tool for detecting central scotomas and metamorphopsia, and study has shown that abnormalities on this test are consistently correlated with dengue maculopathy [19]. Serial microperimetry is preferred for monitoring dengue-related maculopathy with central scotomata, as it offers greater sensitivity and can detect reductions in scotoma size that are not observable with Goldmann perimetry [64]. Perimetry can identify central scotomas and additionally detect peripheral visual field defects, which may be associated with conditions like vasculitis or optic neuritis. Both central and peripheral findings are valuable for monitoring disease progression through serial scotoma mapping [33].

### 6.3. Optical Coherence Tomography (OCT)

**Figure 3:**
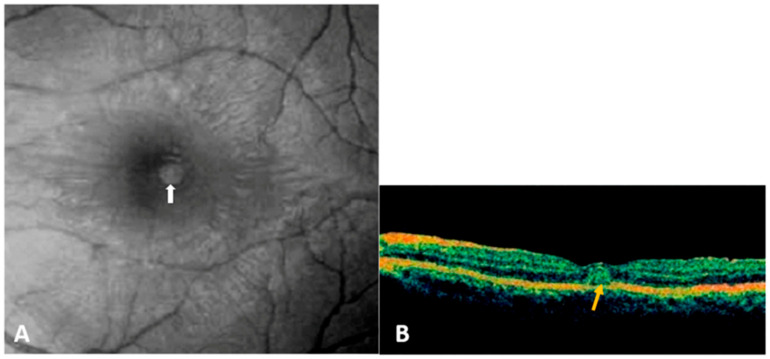
(**A**) Red-free fundus photograph of the left eye in a patient with dengue fever showing a round, yellowish subretinal lesion at the fovea (white arrow). (**B**) Optical coherence tomography scan through the lesion reveals a conical foveal elevation (yellow arrow) associated with focal thickening of the outer neurosensory retina and retinal pigment epithelium. (Adapted with permission from [65] 2023 Zina et al.).

OCT serves as an indispensable tool for evaluating macular health, particularly for assessing retinal thickness and detecting edema in patients with maculopathy (Figure 3) [33]. A meta-analysis including 778 records showed that OCT findings were observed in 67.09% (CI, 35.65–88.24) patients with ocular manifestations in dengue fever, while blurred vision and scotomas were reported in 55.5% (CI, 25.08–82.33) and 36.2% (CI, 15.86–62.99) of cases [14].

Three distinct OCT patterns of dengue-related maculopathy have been described, each correlating with unique structural characteristics, clinical presentations, and prognostic implications [24]. (1) Type 1, or diffuse retinal thickening, is the most observed pattern and is characterized by increased retinal thickness around the central or paracentral fovea, sometimes accompanied by loss of the foveal dimple. Patients with Type 1 typically experience only mild reductions in visual acuity, with over 85% maintaining better than 6/12 vision. It carries the most favorable prognosis, with more than 60% of patients experiencing resolution of relative scotomas within two years. (2) Type 2, CME, presents with sudden visual loss and marked intraretinal cystic changes extending to the photoreceptor layer, appearing as ovoid hyporeflective spaces with intervening septa on OCT. Although macular thickness usually recovers within six months and 81% retain good visual acuity at two years, more than half experience residual scotomata. (3) Type 3, foveolitis, is defined by localized thickening and hyperreflectivity in the subfoveal outer retina, corresponding to disruption of the inner segment/outer segment (IS/OS) junction. This type is associated with the worst visual outcomes, presenting with poor visual acuity and persistent paracentral or central scotomata that may remain despite resolution of retinal edema and restoration of anatomical structure.

### 6.4. Fundus Fluorescein Angiography (FFA)

Fundus Fluorescein Angiography (FFA) serves as a valuable diagnostic tool for assessing inflammatory activity and retinal perfusion in dengue uveitis [66]. It is particularly useful in diagnosing retinal vasculitis, where suggestive findings include arteriolar and venular leakage, early-phase hypofluorescence (often due to masking from intra-arterial retinal hemorrhages), late-phase vessel wall hyperfluorescence, delayed vessel filling, and optic disc staining caused by dye extravasation [19,20,33,44,45]. However, despite its utility in vasculitis, the sensitivity and overall utility of FFA in diagnosing dengue maculopathy may be limited. For instance, in one study focusing on dengue maculopathy, only three of the ten most severely affected eyes showed significant FFA changes, specifically early foveal hyperfluorescence persisting into the late phase, which correlated with worse visual acuity at presentation [67].

### 6.5. Indocyanine Green Angiography (ICG-A)

Indocyanine Green Angiography (ICG-A) offers advantages over FFA, particularly in detecting foveolitis and certain paramacular lesions associated with dengue. Yellow-white paramacular lesions, which might be unremarkable or missed on FFA, can be clearly detected by ICG-A [33]. Bacsal et al. attributed these findings to involvement of the choriocapillaris–RPE complex, though it remained uncertain whether the lesions were inflammatory or occlusive in origin [19,33,64].

### 6.6. Optical Coherence Tomography Angiography (OCTA)

Optical coherence tomography angiography (OCTA) detects alterations in vessel density within the superficial and deep capillary plexuses and may be more sensitive than FFA for identifying subclinical microvascular ischaemia. OCTA findings have been well characterized in both dengue maculopathy and AMN [23,66,68].

## 7. Treatment

The management of dengue-related ocular complications involves a balance between active surveillance and various therapeutic interventions, primarily corticosteroids and, in resistant cases, intravenous immunoglobulin.

### 7.1. Vaccines and Prevention

At present, two dengue vaccines are licensed—CYD-TDV (Dengvaxia) and TAK-003 (Qdenga); TV003 (Butantan-DV) remains investigational rather than licensed [4]. To date, no clinical trials have specifically evaluated strategies to prevent ocular involvement in dengue or whether vaccination reduces the risk of such complications.

### 7.2. Active Surveillance

Several reports have documented spontaneous recovery in dengue-related ocular complications. Bascal et al. adopted active surveillance as the preferred management strategy for patients with maculopathy who presented with good initial visual acuity, subjective improvement, or contraindications to steroid therapy [33]. These patients were followed for 1 to 14 weeks, though complete resolution was not always specified [33]. Chan et al. observed a median recovery time of 3 days without treatment in 11 patients with suspected dengue maculopathy [10]. Similarly, Chlebicki et al. reported that 3 of 4 patients with macular hemorrhages showed symptomatic improvement within 2 days, though one had incomplete recovery after 2 months [69]. Loh et al. described a patient with foveolitis and branch venular occlusion who improved to 20/30 vision within 3 days and reached 20/20 by 3 weeks [67]. Similarly, Tabbara noted spontaneous resolution within 6–8 weeks in two cases of multifocal chorioretinitis [44]. These instances suggest that a self-limiting course is possible for some ocular manifestations.

### 7.3. Corticosteroid Therapy

Despite the self-limiting nature of many ocular manifestations, corticosteroids are frequently employed, though their efficacy and optimal dosing remain unproven due to the lack of randomized controlled trials (RCT). It is often unclear whether recovery is due to natural disease progression or the intervention itself [55].

Topical corticosteroids were commonly used in cases of anterior uveitis and acute primary angle closure. Teoh et al. reported successful treatment of 5 patients with anterior uveitis using topical prednisolone 1%, achieving resolution by day 7 without relapse after tapering [42]. Other local steroid therapies included periocular methylprednisolone [33], subconjunctival dexamethasone, sub-Tenon’s triamcinolone [13], and intravitreal triamcinolone acetonide [33].

Systemic corticosteroids were preferred mainly in cases with sight-threatening presentations. Chan et al. reported one patient given oral prednisolone 1 mg/kg/day for a week, tailed off slowly over 2 months and one patient given intravenous methylprednisolone 250 mg for 3 days. Both of them demonstrated visual recovery with resolution of clinical signs after 1 month with no reported adverse effects [10]. Teoh et al. used a similar steroid regimen in 13 cases of extensive panretinal vasculitis and exudative retinal detachment, one with optic neuritis [42]. All patients presented with a best-corrected visual acuity (BCVA) worse than 20/200 and were treated with systemic steroids without adverse effects [42]. Among them, 7 patients (11 eyes) who received intravenous methylprednisolone (250 mg every 6 h for 3 days), followed by oral prednisolone (1 mg/kg/day for 1 week), achieved BCVA ranging from 20/40 to 20/200 after one year [42]. In contrast, those treated with oral steroids alone showed recovery outcomes similar to untreated patients [42].

Steroid therapy was sometimes withheld due to concerns about side effects. Periocular corticosteroids were avoided in cases with low platelet counts, and systemic steroids were not used when an infectious etiology was suspected [33]. In one case, systemic steroids were discontinued due to steroid-induced glaucoma, necessitating a transition to steroid-sparing agents [70].

### 7.4. Intravenous Immunoglobulin (IVIG)

Intravenous immunoglobulin (IVIG) is typically considered when patients show insufficient improvement after initial high-dose intravenous methylprednisolone. Bacsal et al. described a patient with poor improvement after three days of intravenous methylprednisolone (1 g/day), who was subsequently treated with IVIG at 400 mg/kg over five days, resulting in visual improvement from counting fingers to 6/60 [33]. Similarly, Chang et al. reported a case of retinal vasculitis unresponsive to two days of intravenous methylprednisolone (IVMP), in which IVIG was administered followed by IV hydrocortisone, leading to rapid visual recovery from 6/120 to 6/15 within six days. The patient was then discharged on a tapering dose of oral prednisone [12]. These cases suggest IVIG can be an effective rescue therapy for severe, refractory dengue-related ocular inflammation. However, a case has been reported in which the patient’s vision failed to improve despite IVIG therapy [54].

### 7.5. Surgical Management

Surgeries were dependent on the clinical presentation and were adjunctive. Pars plana vitrectomy was performed in patients with vitreous hemorrhage [71,72]. Peripheral iridotomy with ocular hypotensives was used for acute angle closure [73], and panretinal photocoagulation for retinal hypoperfusion with pre-retinal hemorrhage to prevent neovascularization [74]. Bilateral glaucoma valve implantation for patient with secondary glaucoma in panuveitis is also reported [70].

## 8. Prognosis

While many patients with dengue-related ocular complications achieve full spontaneous recovery, it is crucial to acknowledge that some severe cases can result in blindness or long-term persistent scotomas. For example, in a case series, all patients were observed to attain final Snellen visual acuity of at least 6/9, except one patient whose visual acuity remained at his baseline of 6/12 [20]. However, in some cases of dengue-related maculopathy, scotoma could persist for a long time despite VA improvement [56,75].

Visual outcomes depend on initial severity. Poor visual outcomes in dengue-related ocular disease are often associated with severe initial presentations or profoundly reduced baseline visual acuity. Bawankar et al. described a patient who presented with no perception of light (NPL) and showed no improvement despite treatment [35]. Similarly, according to the OCT-based classification by Teoh et al., type I diffuse retinal thickening had the most favorable prognosis, with a 2-year mean BCVA of 20/25 and nearly 70% resolution of scotomata [24]. Type II cystoid macular edema shared a similar visual outcome, but with a lower rate of scotoma resolution (43.8%) [24]. Type III foveolitis, despite clinical and anatomical recovery, showed persistent scotomata at 2 years [24].

## 9. Conclusions

Ocular manifestations of dengue infection are no longer considered rare, presenting a significant aspect of the disease’s overall impact. The primary ophthalmic complications include uveitis, maculopathy, and various forms of hemorrhage, all largely understood to be driven by immune-mediated pathogenesis. Diagnostic investigations are tailored to the clinical presentation, often encompassing OCT, FFA, and ICG to characterize the extent and nature of the ocular involvement.

Currently, a significant challenge in managing these complications is the absence of RCT evaluating treatment efficacy, meaning standardized treatment protocols are lacking. Consequently, most patients are managed either through close observation, particularly for self-limiting conditions, or with anti-inflammatory therapies such as corticosteroids or IVIG. However, the precise benefit of these interventions remains unclear, underscoring the critical need for further research to establish evidence-based guidelines.

The prognosis for dengue-associated ocular disease is highly variable, ranging from full recovery to cases of persistent visual loss and residual scotomas. Despite this variability, the overall outlook is generally favorable, with a considerable number of patients successfully regaining their baseline visual acuity.

## Data Availability

Not applicable.
